# Active and passive stabilization of body pitch in insect flight

**DOI:** 10.1098/rsif.2013.0237

**Published:** 2013-08-06

**Authors:** Leif Ristroph, Gunnar Ristroph, Svetlana Morozova, Attila J. Bergou, Song Chang, John Guckenheimer, Z. Jane Wang, Itai Cohen

**Affiliations:** 1 Department of Physics, Cornell University, Ithaca, NY 14853, USA; 2 School of Applied and Engineering Physics, Cornell University, Ithaca, NY 14853, USA; 3 Department of Mathematics, Cornell University, Ithaca, NY 14853, USA; 4 School of Mechanical and Aerospace Engineering, Cornell University, Ithaca, NY 14853, USA; 5 IJK Controls, Dallas, TX 75231, USA; 6 Department of Engineering, Brown University, Providence, RI 02912, USA

**Keywords:** insect flight, flapping flight, stability, control, fruit fly, flight dynamics

## Abstract

Flying insects have evolved sophisticated sensory–motor systems, and here we argue that such systems are used to keep upright against intrinsic flight instabilities. We describe a theory that predicts the instability growth rate in body pitch from flapping-wing aerodynamics and reveals two ways of achieving balanced flight: active control with sufficiently rapid reactions and passive stabilization with high body drag. By glueing magnets to fruit flies and perturbing their flight using magnetic impulses, we show that these insects employ active control that is indeed fast relative to the instability. Moreover, we find that fruit flies with their control sensors disabled can keep upright if high-drag fibres are also attached to their bodies, an observation consistent with our prediction for the passive stability condition. Finally, we extend this framework to unify the control strategies used by hovering animals and also furnish criteria for achieving pitch stability in flapping-wing robots.

## 1. Introduction

Flight of both animals and machines requires not only generating aerodynamic force sufficient to overcome gravity but also maintaining balance while aloft [1–3]. For fixed-wing aircraft, the need for balance has led to solutions ranging from passenger airliners that are stable by design to fighter jets that require active control of wing surfaces to overcome intrinsic instabilities [4]. More generally, control strategies are constrained by instabilities, with fast-growing instabilities demanding fast reactions [5,6]. Here, we show how these same principles play out in the case of flapping-wing flight of insects. We apply techniques from dynamical systems and control theory to form a framework that links the physical stability characteristics of flying insects with the sensory–motor systems needed for control. We use the fruit fly, *Drosophila melanogaster*, as a model organism in establishing this framework and also show how the stabilization strategies of other animals and flapping-wing robots can be assessed.

Our approach builds on the last decade's rapid progress in understanding how intrinsic stability or instability emerges from the aerodynamics of flapping wings. A variety of studies have used computational fluid flow solvers [7–10] and aerodynamic models [11–13] to assess a simulated insect's passive dynamical response to flight perturbations. Such studies have shown that the back-and-forth flapping motions characterizing the wing kinematics of fruit flies and a broad class of other insects induce an oscillating diverging instability in the body pitch orientation. The appearance of this instability in a variety of simulations using different body plans and wing motions suggests that it is a generic feature of insect flight. Thus, the pitch dynamics is an appealing platform for investigating how insect flight control systems contend with physical instabilities.

The intrinsic instability of body orientation in flapping-wing flight seems consistent with the highly specialized sensory adaptations of flying insects [14]. The flight control system of the true flies (order *Diptera*), for example, includes the halteres, a pair of stalk-like organs that flap and serve as vibrating structure gyroscopes capable of sensing body rotations [15,16]. The sensory neurons at the base of the halteres are directly wired to the motor neurons of the flight muscles [17], implicating this system in fast flight behaviours. Indeed, neural recordings show that the delay or reaction time of the haltere system is fast compared with other sensory modalities such as the visual system [18,19]. These findings, as well as behavioural experiments that apply perturbations to insects [20,21], indicate that the haltere system plays a central role in fast flight stabilization.

In this work, we explore the hypothesis that the flight control systems of insects have evolved to overcome the intrinsic instabilities of flapping flight. Building on the recent work on the role of control delay in animal movement [22–24], we conjecture that the reaction time of sensory–motor flight control systems must be fast relative to the time scale of instability growth. We first motivate this hypothesis through a series of experiments that mechanically perturb the free flight of fruit flies and assess the flight of these insects after manipulations to their sensors and to their body morphology. We then show how these observations can be organized by a control theoretic framework that evaluates flight performance as a function of the physical time scales associated with stability as well as the reaction time of the control system. Finally, we generalize our results to unify the stability and control strategies used by a broad class of hovering insects, hummingbirds and flapping-wing robots.

## 2. Experiments

### 2.1. Flight perturbations

To determine the flight control strategy used by fruit flies (*D. melanogaster*), we apply impulsive torques to these insects while capturing high-speed video of their flight [21,25]. In these experiments, we first glue a small ferromagnetic pin to the dorsal thoracic surface of each insect. We then release many prepared flies within a clear chamber that is placed at the intersection point of three orthogonal high-speed video cameras. Our set-up includes a laser trigger in which a flying insect initiates both video capture as well as the application of an impulsive magnetic field generated by Helmholtz coils. In this study, the coils are placed above and below the chamber, generating a vertical magnetic field and thus a torque that perturbs the body pitch orientation. Nose-up and nose-down perturbations are achieved by running experiments at both polarities of the applied current through the coils. The magnitude of the current, and thus field strength, is selected by trial and error to produce moderate body pitching of 5–25°. Finally, we use custom motion-tracking algorithms to extract the wing and body kinematics from the videos [26], thus providing a window into the flight control response of these insects.

**Figure 1.**
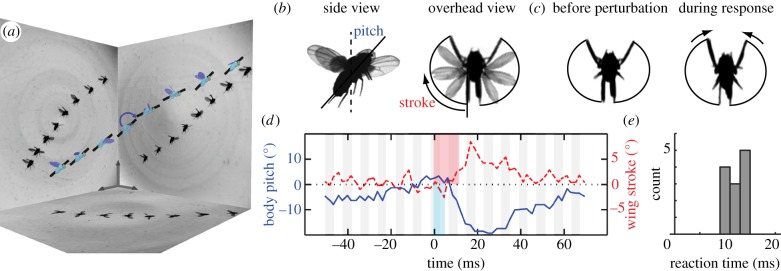
Fruit flies quickly overcome in-flight perturbations. (*a*) Reconstruction of a flight perturbation filmed with three high-speed cameras. Selected images are shown on the side panels, and the measured configurations of the insect (body length 2.7 mm) are displayed on the model. A black bar on the insect body highlights its pitch orientation. As the insect ascends from left to right, an impulsive magnetic field (blue arrow) induces a nose-down torque on the ferromagnetic pin glued to its back. (*b*) Perturbations are applied to the body pitch orientation, and the insect responds with changes to the wing-stroke angle. (*c*) By sweeping its wings further in front, the insect generates a nose-up corrective torque. (*d*) Body pitch (solid blue line) and wing-stroke (dashed red line) angles, with each quantity shifted so that the average pre-perturbation value is zero. The magnetic torque perturbation (thin blue stripe) tips the insect downwards, and the insect responds by correcting its orientation. After a reaction time of 12 ms (thick red stripe), the fly generates corrective wing motions. Each gray and white stripe denotes a wing beat, with a typical period of about 4 ms. (*e*) Histogram of reaction times measured in 12 perturbation experiments. (Online version in colour.)

A typical flight perturbation experiment is shown in the reconstruction of figure 1*a*. Selected images captured from each high-speed camera are shown on the side panels, and the extracted body and wing configurations are displayed on the model insect. A black cylinder is drawn through its long axis, highlighting the changes to body pitch orientation. As the insect ascends and progresses left to right, the magnetic torque is applied (curved arrow) and causes a nose-down rotation. The insect then recovers its pitch orientation by a nose-up rotation as it continues on its trajectory. We quantify these dynamics by measuring the pitch angle over time, and in figure 1*d* we display the deviations in the pitch relative to its mean value prior to the perturbation. The magnetic torque is applied for one wing beat (blue stripe) starting at time zero, and the body pitch then rapidly decreases by almost 20°. Pitch then increases to near its original value, making a recovery in about 60 ms or 15 wing beats.

To gain insight into the fly's stabilization mechanism, we compare the wing motions before the perturbation and during the nose-up response. This recovery is driven by subtle but stereotyped wing adjustments, most notably by increasing the forward sweep of the wings, as shown by the overhead views in figure 1*c*. This observation is consistent with a strategy in which the aerodynamic centre is shifted forwards in order to generate a nose-up pitch torque [27]. To quantify this response, we measure the stroke angle of the wings, and in figure 1*d* we display the deviation in this angle from its value prior to the perturbation. The fast response is characterized by an increase in the stroke angle that occurs about two or three wing beats after the initiation of the perturbation (red stripe). We quantify this reaction time, *T*_RXN_ = 12 ms, by determining the first time in which the stroke angle exceeds and remains higher than any pre-perturbation level.

By conducting many such experiments, we find that the flight control response of fruit flies to pitch perturbations is similar across different individuals. In particular, for 12 trials of near hovering flight—with the body speed always less than 20 per cent of the wing speed—we measure that the mean reaction time is *T*_RXN_ = 13 ± 2 ms, as shown by the histogram in figure 1*e*. We note that our determination of the reaction time reflects the entire behavioural response sequence. Thus, in addition to the neural delay time that has been measured to be 2–9 ms [19], our measurements also include the physical lag times associated with body motion to sensor detection and muscle activation to wing motion. Further, our measurements for pitch control are in quantitative agreement with the delay in wing adjustments for fruit flies responding to yaw perturbations [21]. Thus, these insects are able to remain upright against flight disturbances by applying fast, active control over wing motions.

### 2.2. Manipulation of sensors and body plan

**Figure 2.**
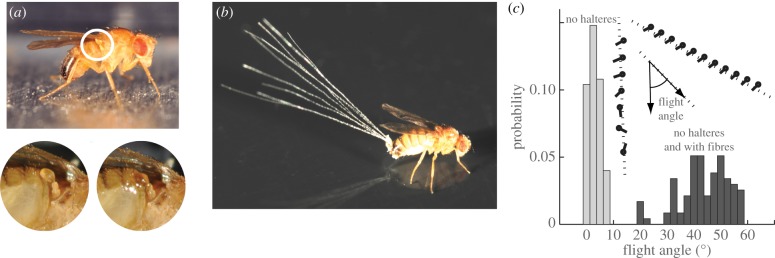
Active and passive stabilization of fruit fly flight. (*a*) Fruit flies use fast gyroscopic sensors called halteres to mediate flight control. Each haltere vibrates during flight and detects changes in body orientation. If glued down, the haltere no longer properly functions. (*b*) Dandelion seed fibres add drag to the insect body, thus increasing passive stability. (*c*) Inset: body orientation and flight trajectory of a fly with halteres disabled (left), showing a tumbling motion while falling downwards. When fibres are attached to a haltere-disabled insect, it is able to keep upright as it descends (right). Main figure: insects are released in air, and flight performance is assessed by measuring the trajectory angle with respect to the downward vertical. Distributions of flight angles for insects with halteres disabled (light grey) and insects with halteres disabled and with fibres attached (dark grey). (Online version in colour.)

To offer further evidence that fruit flies rely on active control, we revisit classic experiments that disable the fast mechanical sensors of these insects [1,15,16]. As shown in figure 2*a*, the halteres of the fly are located below each wing and oscillate in flight, serving as gyroscopic sensors of body rotations. Here, we disable this sensory function by glueing the halteres to the abdomen and thereby preventing any oscillations. When released in still air, these sensor-disabled insects fall nearly straight down as indicated by the measured left trajectory in figure 2*c*. High-speed video shows that these flies are indeed flapping their wings at typical frequencies and amplitudes but that their body rapidly tumbles nonetheless, suggesting that a lack of orientational control undermines their flight. To quantify their flight performance, we release sensor-disabled flies from 1 m high and measure the radial distance these insects are able to travel. We then compute the flight angle as the inverse tangent of the ratio of the distance travelled to the initial drop height. A histogram of many trials is shown in light grey in figure 2*c*, and the typical flight trajectory angle near zero quantifies their poor flight performance.

Interestingly, stability can be reinstated by modifying the insect body itself [15,16]. In these experiments, we glue down the halteres and also attach to the abdomen approximately 10 thin fibres taken from a dandelion seed pod, as shown in figure 2*b*. When released in air, these modified insects are able to maintain a coherent flight trajectory, typically descending at a flight angle near 45° before landing on the ground. This increased performance is quantified in figure 2*c* by the dark grey histogram of flight angles for many trials. Further, a typical flight trajectory extracted from high-speed video is shown to the right in the inset of figure 2*c*, revealing that these flies are able to maintain an upright orientation throughout flight. Thus, while these insects are rather weak flyers, it appears that this modification enhances orientational stability and allows for directed flight over long distances.

Collectively, these experimental observations suggest that the flapping-wing flight of insects is intrinsically unstable but may be either actively controlled with fast reactions or passively stabilized with high body drag. In the following section, we use these findings as a guide for formulating a mathematical model that unifies the elements of stability and control.

## 3. Model of pitch stability and control

### 3.1. Body pitch dynamical model

Recent aerodynamic simulations indicate that body orientation is intrinsically unstable for some insects [7,8,11,13]. These simulations include both computational fluid dynamics (CFD) codes that numerically solve the Navier–Stokes equations as well as quasi-steady models that approximate aerodynamic forces on the flapping wings. Both formulations couple fluid forces to the rigid-body dynamics of the insect body, and simulations of a variety of flies, bees and moths show that body pitch is subject to diverging oscillations.

**Figure 3.**
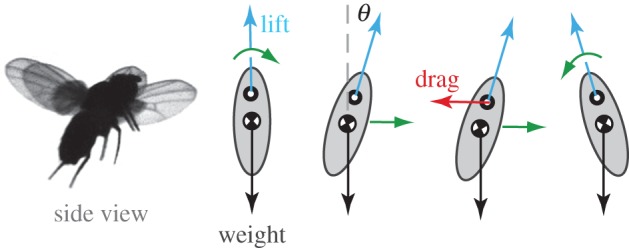
A force diagram shows how an instability in body pitch develops. During hovering, the wings flap back and forth to produce lift that balances body weight. A perturbation to the pitch orientation causes the insect to move forwards. Drag on the wings then becomes unbalanced, inducing a torque that tends to tip the insect backwards. This simplified picture highlights some of the important effects to be incorporated in a model of the pitch dynamics. (Online version in colour.)

These simulations motivate a simplified physical picture that captures the nature of the instability [7]. As shown in figure 3, the relevant physical mechanisms can be illustrated most simply for normal hovering flight. The wings beat back and forth, the average lift is directed upwards to balance body weight, and drag points horizontally but cancels for the two half-strokes. If pitched forwards, the re-directed lift drives the insect forwards. This leads to a net drag on the wings, because the wing airspeed on the forward sweep is now greater than the airspeed on the backward sweep [28–30]. If the wings are located above the body centre of mass, as is the case for flies and many other insects [31], this drag creates a nose-up pitch torque that rotates the insect. In effect, the insect is flipped backwards by the drag on its wings. If left uncontrolled, this instability rocks the insect back and forth with growing amplitude and ultimately causes it to tumble from the air.

These observations suggest a minimal set of ingredients to be included in a reduced-order model. The insect body is an extended rigid body of mass *M* and pitch moment of inertia *I*. We will evaluate longitudinal motion of this body, that is, the dynamics of the forward velocity, pitch angle and pitch rate (*u*,*θ*,*ω* = *θ*). The body orientation during hovering defines *θ* = 0. So-called ‘normal hovering’ insects beat their wings back and forth [32], and the average lift points upwards and balances body weight: *L* = *Mg*. We assume that the wings co-move with the body, so the average lift vector is of fixed position and orientation with respect to the body [31,33]. As we will show, the distribution of drag is critical to the flight dynamics. For the sake of generality, we assume that the insect body has distributed sources of linear drag ***D*** at displacements ***r*** from the centre of mass. Such drag sources include the wings, which have been shown to give rise to drag linear in body velocity [30], as well as viscous forces on the body itself and on other damping surfaces such as the legs. Drag is directed opposite to the velocity ***u*** for each drag source, d***D*** = −***u*** · d*μ*. Here, d*μ* represents the dependence of drag on aerodynamic characteristics of the source, such as size and coefficient of drag, as well as on fluid properties, such as density and viscosity.

We next write down a general linear model of the longitudinal dynamics. If the insect is pitched, horizontal body acceleration 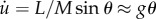 results from the re-directed lift. The vertical body acceleration stemming from the loss of lift is a second-order effect and therefore not included in this analysis. Drag causes both translational and angular velocities to couple to both translational and angular accelerations. Thus, the system can be described using the linearized Newton–Euler equations,3.1
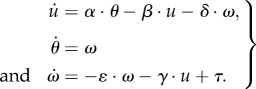
Here, *α* = *g* is the re-directed lift-based acceleration, the coefficients (*β*,*γ*,*δ*,*ɛ*) define how the sources of drag give rise to accelerations and *τ* is the net external pitch torque divided by the body moment of inertia. Evaluation of the drag sources distributed at displacements ***r*** from the body centre of mass leads to the following relations: 
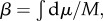
 
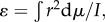
 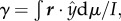 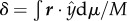 with 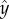 the vertical unit vector. Thus, coefficients in this dynamical system appear as ratios of moments of the drag distribution and moments of the mass distribution, 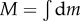 and 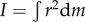.

We note that our simplified model of the pitch dynamics is similar in form to those presented in previous studies [7,11,13,34]. Our goal is to re-cast these equations in terms of the relevant time scales, a language that offers physical intuition and lends itself to biological interpretation.

### 3.2. Time-scale formulation of the model

Algebraic elimination of the dynamical variables *u* and *ω* in favour of the pitch angle *θ* leads to a third-order differential equation that describes pitch in the absence of external torques, *τ* = 0:3.2
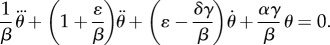


Thus, while the variables *β* and *ε* appear on their own, the other variables appear only in combinations. To physically interpret these combinations, it is convenient to define a centre-of-drag vertical displacement from the centre of mass, 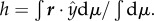 Then, one can show that *αγ*/*β* = *Mgh*/*I* and *δγ*/*β* = *Mh*^2^*β*/*I*. This second term is negligible for small *h*, that is, when drag sources are distributed nearly symmetrically about the body.

The dynamics of equation (3.2) lends itself to an interpretation in terms of physical time scales. First, forward motion is damped with a characteristic time of *T*_F_ ≡ 1/*β*. Likewise, pitch rotations are damped over a time scale of *T*_P_ ≡ 1/*ε*. Finally, the insect body has an inherent inertial rotation time scale of 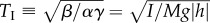, which is similar in form to the oscillation period of a compound pendulum supported a distance *h* from its centre of mass. Cast in these variables, the pitch dynamical equation becomes3.3
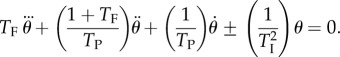


Here, the positive form refers to a low centre of mass relative to centre of drag, while the negative form refers to top-heavy body plans. This equation is similar to a damped rotational oscillator. In fact, in the limit of strong forward damping, *T*_F_ → 0, the dynamical equation has the same form as that of a damped compound pendulum. But the presence of forward damping leads to two differences. First, the effective moment of inertia is enhanced by the ratio *T*_F_/*T*_P_. Second and most critically, the presence of the third-derivative term acts as a source term that injects energy into the oscillator and can destabilize it.

### 3.3. Assessing flight stability

The inherent stability of body pitch during flight can be formally evaluated by assessing the mathematical stability of equation (3.3). This procedure considers solutions of the form *θ* ∼ e*^*λ*t^*, where *λ* is a complex eigenvalue of the system [6]. This leads to the characteristic equation3.4
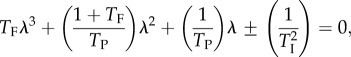
which has three roots corresponding to three eigenvalues. If the real part of any root is greater than zero, then deviations grow exponentially and the system is unstable. If all eigenvalues have negative real parts, then deviations decay and the system is stable.

The Routh criterion provides a general relationship among the physical time scales necessary to furnish inherent stability [6]. If the centre of drag is low, corresponding to the negative form of equation (3.3), then this criterion indicates that flight is unstable for all values of the physical time scales. If the centre of drag is high, corresponding to the positive form of equation (3.3), then the Routh criterion for stability is3.5
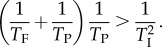


If equation (3.5) is not satisfied, then flight is unstable and pitch perturbations grow exponentially in time. In this case, the time scale of instability growth, *T*_INST_, is related to the eigenvalue of equation (3.4) with positive real part and thus can be computed from the physical time scales.

**Figure 4.**
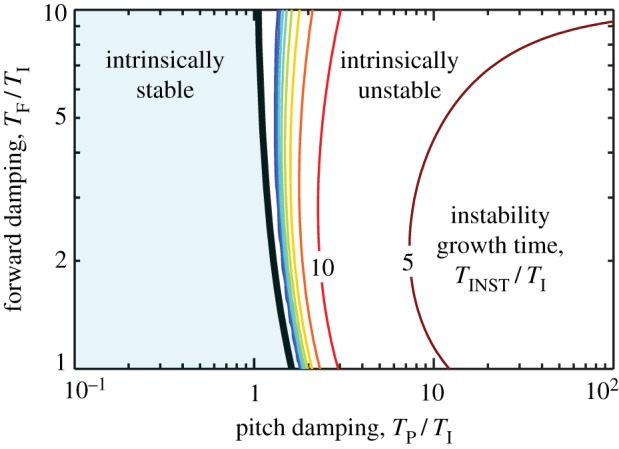
Stability diagram for insect flight. Stability characteristics are plotted as a function of dimensionless forward and pitch damping time scales, *T*_F_/*T*_I_ and *T*_P_/*T*_I_. The light blue region to the left of the black line corresponds to intrinsic stability. The region to the right is unstable, and the contours show the dimensionless instability growth time, *T*_INST_/*T*_I_. (Online version in colour.)

These aspects of intrinsic stability can be summarized by the diagram shown in figure 4. Here, we have non-dimensionalized all relevant time scales by the inertial time *T*_I_. First, we plot the stability criterion of equation (3.5) as the heavy black curve. To the left of this stability boundary (blue region), the dimensionless damping time scales *T*_F_/*T*_I_ and *T*_P_/*T*_I_ are such that flight is intrinsically stable. To the right, flight is unstable and the coloured contours represent the dimensionless instability growth time scale, *T*_INST_/*T*_I_. Near the boundary, it grows slowly, and as one moves to the right on the diagram there is a broad region in which the instability grows a few times faster than the inertial time scale.

This diagram shows that the stability properties depend only weakly on the parameter *T*_F_/*T*_I_: neither the intrinsic stability boundary nor the instability growth time vary strongly with forward damping. Thus, in much of the remaining analysis, we will hold *T*_F_/*T*_I_ = 5.1, which we calculate as the value of this parameter for fruit flies. However, the parameter *T*_P_/*T*_I_ is critical to determining intrinsic stability. In particular, the stability boundary is located near *T*_P_/*T*_I_ ≈ 1. Thus, when body rotations are damped quickly compared with inertia, flight is passively stable. When rotations are weakly damped relative to inertia, flight is unstable and active control is necessary.

### 3.4. Active control and reaction time

We next seek a quantitative framework that describes the demands placed on the active control system by the presence of the pitch instability. Just as the physical aspects of stability can be written in terms of time scales, a flight control scheme can be characterized by its delay or reaction time, *T*_RXN_. Our formulation is guided by the intuition that unstable processes are inherently difficult to control, especially in cases in which the control response is delayed in time [5]. In such cases, control theory provides rules of thumb for assessing performance [6,35,36]. Perhaps the simplest rule is that reactions must be several times faster than the growth of the instability. This suggests that the time-scale ratio *T*_INST_/*T*_RXN_ is a natural performance metric, with higher values indicating better control.

**Figure 5.**
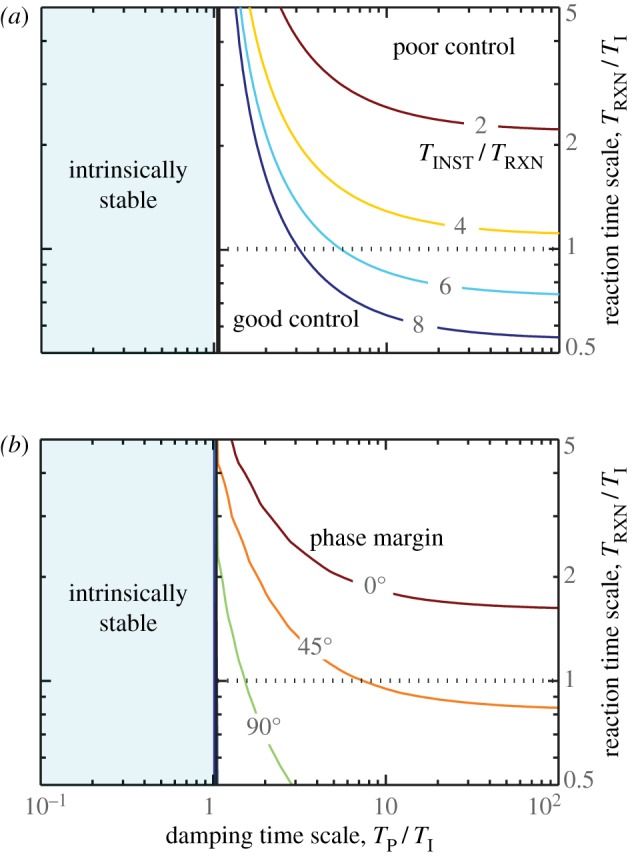
Stability and control properties of insect flight. Dimensionless reaction *T*_RXN_/*T*_I_ versus pitch damping *T*_P_/*T*_I_ time scales for fixed *T*_F_/*T*_I_ = 5.1, as appropriate for a fruit fly. The light blue regions indicate intrinsic stability due to strong damping, and the white region indicates intrinsic instability that may be controlled with fast reactions. (*a*) Coloured contours correspond to a control performance metric of the ratio of instability to reaction time scales. Good control (blue) is achieved by strong damping or fast reactions, while poor control (red) corresponds to weak damping and slow reactions. (*b*) Coloured contours correspond to a control performance metric of the phase margin. Generally, good performance is achieved with a phase margin of at least 45°. (Online version in colour.)

The passive and active aspects of flight stabilization are then unified in figure 5*a*, which plots contours of this performance metric as a function of the dimensionless pitch damping and reaction times. Qualitatively, one can think of the horizontal axis as indicating decreasing passive stabilization, the vertical axis as indicating decreasing active stabilization, and the contours as the overall control performance. Here, a given insect should be viewed as a set of four independent time scales, namely the three physical time scales (*T*_I_,*T*_F_,*T*_P_) as well as its reaction time *T*_RXN_. Intrinsic or passive stability is achieved if 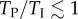 (blue region) and, in this case, the value of *T*_RXN_ is irrelevant since no active control is needed. If this condition is not met, then flight is intrinsically unstable and *T*_INST_ is determined by the three physical time scales. Control performance can then be quantified by the ratio *T*_INST_/*T*_RXN_. For example, a performance of *T*_INST_/*T*_RXN_ = 6 (light blue curve) can be achieved by a family of solutions ranging from slow reactions but high drag (upper left) to fast reactions and low drag (lower right). In general, the value of *T*_INST_/*T*_RXN_ increases as one moves down or to the left, quantifying the better flight control that one expects with faster reactions or increased damping.

Control theory also provides a second and more involved assessment of performance for the delayed control of an unstable system [6,35,36]. For the control theory enthusiast, we present this phase margin analysis below. Importantly, we show that the two metrics give similar results, with acceptable control performance corresponding to a phase margin of about 45° and a time-scale ratio of *T*_INST_/*T*_RXN_ ≈ 6.

The phase loss formulation determines the fundamental limitations placed on control performance by the presence of control delay and physical instabilities [6]. To understand the analysis in the context of this work, consider the pitch torque on the fruit fly as a signal that propagates around a loop [21]. An external torque is the input signal that triggers the unstable pitch dynamics, the resulting pitch rate is then detected by the halteres, and a sensory–neural controller dictates the compensatory torque generated by the wings. This corrective torque is the output signal, and the reaction time *T*_RXN_ is the delay between the input disturbance and this output response.

The key principle is that performance suffers when there is a phase shift, or phase loss, for an input disturbance torque that propagates through the physical process and controller. Consider, for example, how a sinusoidal disturbance torque is affected by different phase losses under the action of negative feedback. For a phase loss of 0, the corrective torque exactly cancels the disturbance signal. A phase loss of *π*, on the other hand, reinforces the disturbance and leads to instability of the combined controller–process system. Thus, controllers and physical processes that induce phase loss are generally undesirable. The phase loss owing to both instabilities and time delays can be rigorously evaluated and then compared with standards for acceptable control performance.

The qualitative reasoning given above has been formalized into the so-called *design inequality* [6,36],3.6
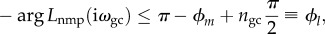
with good performance requiring that the constants *ϕ*_*m*_ ≈ *π*/4 and *n*_gc_ ≈ −0.5 and thus *ϕ*_*l*_ ≈ *π*/2. Here, *L* = *PC* is the open-loop transfer function for a physical process *P* and a controller *C*, and *L*_nmp_ is its so-called non-minimum phase portion, which includes the effects of instabilities and delays. The gain crossover frequency *ω*_gc_ is the frequency for which *|L|* = 1.

A pure delay of *T*_RXN_ corresponds to *L*_nmp_(*s*) = exp(*−s·T*_RXN_), which then gives −arg*L*_nmp_(*s* = i*ω*_gc_) = *ω*_gc_ · *T*_RXN_ [36]. Thus, long delays are troublesome since they correspond to a pure loss of phase.

Instabilities also induce loss of phase, and the loss depends on the nature of the instability or ‘pole’ in the system dynamics. For a pure real pole (or eigenvalue) of *λ* = *σ* > 0, *L*_nmp_(*s*) = (*s* + *σ*)/(*s* − *σ*), which then gives −arg*L*_nmp_(*s* = *i*ω**_gc_) = 2arctan(*σ*/*ω*_gc_) [36]. Thus, fast instabilities (large *σ*) are also troublesome because of the large phase loss.

For the case of insect flight, we typically have a pair of complex conjugate poles, *λ* = *σ* ± *iρ*, with *σ* > 0 signifying an oscillating divergence instability. In this case, the phase loss of each pole can be shown to be 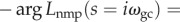 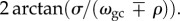 Combining the two poles and delay together gives the following design inequality:3.7
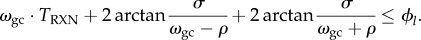


Thus, given *T*_RXN_ as well as the physical time scales *T*_I_, *T*_F_ and *T*_P_ that determine *σ* and *ρ*, one can calculate the phase loss and thus the control performance of the system.

Conventionally, one characterizes the performance by the phase margin *ϕ*_*m*_, which is contained in the phase loss expression of equation (3.6), with *ϕ*_*m*_ ≈ *π*/4=45° denoting acceptable performance [6,36]. In figure 5*b*, we plot contours of the phase margin for varying dimensionless reaction time and pitch damping time. Comparing this plot with figure 5*a*, we see that a phase margin of *π*/4 = 45° is similar to a rule of thumb that *T*_INST_/*T*_RXN_ ≈ 6.

In summary, this phase margin analysis offers a way to assess the performance of a delayed control system that seeks to overcome the pitch instability. The power of this approach is that it does not depend on the details of the control scheme, such as the quantity being sensed or the specific control law implemented. The analysis only requires the physical time scales as well as the reaction time as its input variables. In the following section, we take advantage of this generic formulation to assess the performance not only for fruit flies but also for other animals and flapping-wing robots.

## 4. Results

### 4.1. Estimating physical time scales

To apply this framework to the fruit fly, we must compute the physical time scales *T*_I_, *T*_F_ and *T*_P_. With an eye towards expanding our analysis to include other insects, here we present a general procedure for estimating these time scales. First, the inertial time scale is given by 
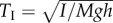
, which depends on the body morphological parameters *M*, *I* and *h*. Body mass *M* is taken from the literature directly, and the moment of inertia is estimated as *I* = *M*(*L*^2^ + 3/4*d*^2^)/12 by approximating an insect's body as a cylinder of length *L* and diameter *d*. The distance from the centre of mass to the centre of drag is *h*, which we approximate as the distance from the centre of mass to the wing attachment point. For the fruit fly, the centre of mass is located near where the abdomen and thorax connect, and we take the centre of drag to be located at the wing attachment point.

The forward damping time scale *T*_F_ is largely determined by wing drag [30]. To evaluate the resistance to forward motion, we approximate the instantaneous drag on the two wings by the high Reynolds number law *D* = 2 × *ρSC*_D_*v*^2^/2, where *ρ* is the fluid density, *S* is the wing area, *C*_D_ is the coefficient of drag and *v* is the wing speed relative to air. For forward flight, the resistive damping force arises from the faster wing speed and thus greater drag on the forward sweep than on the backward sweep. For wings that beat at speed *w* relative to the body and a body moving with speed *u*, the forward dynamics can be determined by averaging the drag for the two half-strokes,4.1
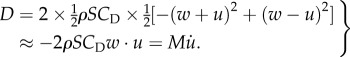


The approximation considers small body speeds and thus drops second-order terms or order (*u/w*)^2^. Thus, the wings act as a source of drag that is linear in body velocity. The forward damping time represents the characteristic time that motion is slowed and is given by4.2
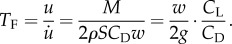


Here, the final equality is a simplification that makes use of the hovering condition that body weight is balanced by lift: *Mg* = *L* = 2 × *ρSC*_L_*w*^2^/2, with a lift coefficient of *C*_L_. Thus, we estimate the forward damping time scale by determining the typical wing speed *w* and lift-to-drag ratio *C*_L_*/C*_D_.

Features other than wing drag may contribute significantly to forward damping. For fruit flies and other small insects in particular, the viscous drag on the body will slow forward motion. Further, for the experiments in which fibres are added to the fruit flies, we expect viscous drag on these structures also to be important. We approximate the drag both on the body and on each fibre by the viscous drag on a cylinder of length *L* and diameter *d* moving at speed *u*: *D* = 4*π*μ*Lu*/ln(*L*/2*d*) [37]. Here, *μ* = 1.8 × 10*^−^*^5^ kg m^−1^ · s is the viscosity of air. In these cases in which multiple sources of damping are important, the cumulative damping time scale must be appropriately computed from all individual sources. For example, if both the wings (W) and body (B) contribute, then 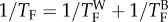.

Pitch damping also arises from different sources, most notably from the wings. During body pitching motions, lift is modified because of changes in the angle of attack [13], although this is a second-order effect for wings that are operating near the angle of maximum lift. Wing drag, however, contributes a first-order effect because of its redirection during pitch rotations. To estimate this contribution, we again consider simplified kinematics consisting of two back-and-forth half-strokes of constant speed *w* = *A/T*, where *A* is the full travel distance of the wing during the wing-beat period *T*. The component of the redirected drag multiplied by the torque arm—which is the wing position *x* from mid-stroke—gives the instantaneous pitch torque,4.3
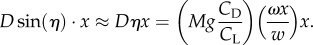


Here, *D* = *L* · *C*_D_/*C*_L_ = *Mg* · *C*_D_/*C*_L_ is the nominal drag, and the drag vector is redirected by an angle *η* ≈ *ωx*/*w* owing to the pitch rotational angular velocity *ω*. Integrating equation (4.3) yields the time-averaged torque over a wing stroke,4.4
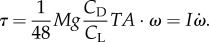


This torque gives rise to the pitch damping time scale of4.5
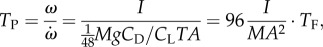
where equation (4.2) is used to yield the second equality. Thus, the wing drag contribution to the pitch damping time scale can be readily estimated from morphological and aerodynamic parameters.

Pitch damping of the fruit fly is also enhanced by viscous drag on the body and, most importantly, by the presence of the fibres. For these elements, we again approximate each structure as a cylinder of length *L* and diameter *d*, and calculate the torque *τ* = *π*μ*L*^3^*ω*/3ln(*L*/2*d*), where *μ* = 1.8 × 10*^−^*^5^ kg m^−1^ s is the viscosity of air [37]. In cases of multiple sources of pitch damping, the cumulative damping time scale is again computed from all individual sources.

### 4.2. Theoretical interpretation of experiments

**Figure 6.**
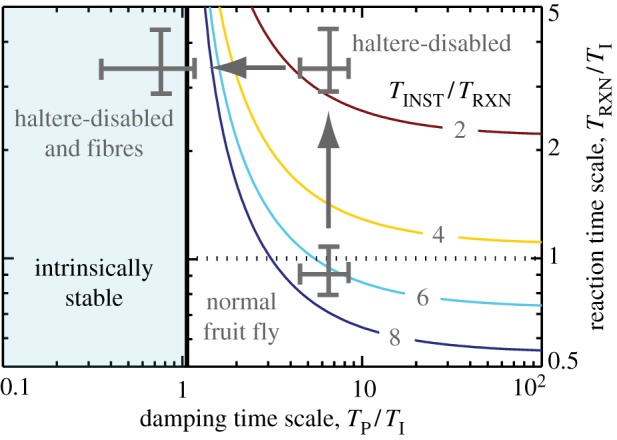
Interpreting experiments on fruit flies using the control and stability diagram. Crosses representing different insects are superposed on the diagram of figure 5*a*. The normal fruit fly is predicted to be intrinsically unstable, and instead relies on reactions that are of the same order as *T*_I_ or about six times faster than the instability, *T*_INST_/*T*_RXN_ ≈ 6. When the halteres are disabled, the insects are left with slower sensors—such as the visual system—and flight performance is significantly degraded. These same insects can be passively stabilized if damping is increased by attaching fibres to their bodies. (Online version in colour.)

To illustrate the utility of the time-scale formulation, we next offer a theoretical interpretation of the experiments. In assessing the flight stability of the fruit fly, we use aerodynamic and morphological parameters and the mean reaction time of *T*_RXN_ = 13 ms measured in the perturbation experiments to determine that *T*_P_/*T*_I_ = 6.1 and *T*_RXN_/*T*_I_ = 0.93, as indicated by the lower cross in figure 6. These data show that fruit flies experience relatively weak damping and thus are inherently unstable. However, these insects are able to actively control flight because of their fast reactions. Consistent with control theory rules of thumb, we find that fruit flies use reactions that are about six times faster than the instability growth time, which is equivalent to a phase margin near 45°.

According to our model, increasing the reaction time of fruit flies would significantly degrade their ability to maintain stable flight. We interpret our experiments in which we glue down the halteres as forcing these insects to rely on slower sensory modalities, such as the visual system which has *T*_RXN_ ≈ 50 ms [18]. Our model indicates that these slower sensors yield significantly worse performance, as indicated by the upper right cross in figure 6. As predicted, the haltere-disabled fruit flies lose control of flight and tumble from the air, as indicated by the flight angle measurements in figure 2*c*.

Interestingly, our formulation predicts that flight capability can be reinstated if pitch damping is increased. In particular, if damping is strong enough such that *T*_P_/*T*_I_ < 1, then flight can be stabilized passively, without the need for active control. In the experiments in which we add fibres to the insects, we estimate that the fibres add so much pitch damping as to cause *T*_P_/*T*_I_ = 0.63 and flight is predicted to be passively stable, as shown by the upper left cross in figure 6. Consistent with our prediction, these insects are able to maintain body orientation while aloft, as indicated by the measurements presented in figure 2*c*.

### 4.3. Generalization to other insects

More broadly, this framework for understanding passive and active flight stabilization is readily extended to other hovering insects, hummingbirds, and even flapping-wing robots. The calculations above allow us to estimate the physical time scales given relatively few parameters for these flyers. In table 1, we compile the relevant morphological and aerodynamic parameters from the literature, and in table 2 we display the calculated time scales. The inertial time *T*_I_ has been used to non-dimensionalize the time scales characterizing forward damping *T*_F_, pitch damping *T*_P_, instability growth time *T*_INST_ and the required reaction time *T*_RXN_. For all flyers, the damping time scales include the wing drag contribution. When other additional sources of drag are important in computing *T*_F_ and *T*_P_, the sources are marked with a note in the column labelled ‘drag’ in table 1.

**Table 1.** Morphological and aerodynamic parameters relevant to pitch stability and control for hovering insects, hummingbirds and flapping-wing robots.

	*M* (mg)	*L* (mm)	*d* (mm)	*h* (mm)	*ϕ*_0_ (°)	*f* (Hz)	*R* (mm)	*C*_L_/*C*_D_	drag	*I* (kg m^2^)
honeybee, *Apis mellifera* [31,33]	101.9	15.9	2.7	3.3	66	197	9.8	1.8/2.3 [38]		2.2 × 10*^−^*^9^
bumblebee, *Bombus terrestris* [39]	595	22.3	5.6	6.0	65	167	15.4			2.6 × 10*^−^*^8^
bumblebee, *Bombus hortorum* [31,33]	226	20.7	3.7	3.9	60	152	14.1			8.3 × 10*^−^*^9^
bumblebee, *Bombus lucorum* [31,33]	231	20.7	3.7	3.9	66	140	14.1			8.4 × 10*^−^*^9^
hover fly, *Episyrphus balteatus* [40]	21.8	11.3	2.8	1.3	37	166	9.0			2.4 × 10*^−^*^10^
drone fly, *Eristaltis tenax* [41]	165.9	14.7	4.5	1.2	55	209	11.2			3.2 × 10*^−^*^9^
hawkmoth, *Manduca sexta* male [42,43]	1199	45.6	7.3 [31]	8.7 [31]	57	26.1	47.3	1.2/0.6 [44]		2.1 × 10*^−^*^7^
hawkmoth, *Manduca sexta* female [42,43]	1833	43.5	7.0 [31]	8.3 [31]	59	25.9	51.0	1.2/0.6 [44]		2.9 × 10*^−^*^7^
fruit fly, *Drosophila melanogaster* [45]	1.0	2.8	1.0	0.4	70	225	2.4	1.4/1.5 [46]	body	7.2 × 10*^−^*^13^
parasitic wasp, *Encarsia formosa* [32]	0.025	0.60	0.22	0.1	65	385	0.66		body	8.3 × 10*^−^*^16^
orchid bee, *Euglossa dissimula* [47]	91.0	10	3	1.5	52	214	8.8			8.1 × 10*^−^*^10^
orchid bee, *Euglossa imperialis* [48]	151.7	13	5	2.5	53	189	10.9			2.4 × 10*^−^*^9^
orchid bee, *Eulaema meriana* [47]	819.6	25	6	4.0	53	105	21.1			4.5 × 10*^−^*^8^
march fly, *Bibio marci* female [48]	65.4	17	4.5	2.1	70	100	11.2			1.3 × 10*^−^*^9^
march fly, *Bibio marci* male [48]	26.6	14	3.5	1.5	69	130	9.4			5.1 × 10*^−^*^10^
conopid fly, *Conops strigatus* [48]	27.1	12	2.6	1.4	72	144	7.7			2.6 × 10*^−^*^10^
bluebottle fly, *Calliphora vicina* [48]	62	10	6.2	2.7	69	135	9.2			5.8 × 10*^−^*^10^
black fly, *Simulium* Latreille [48]	0.80	3.8	1.4	0.7	75	183	3.3		body	9.3 × 10*^−^*^13^
crane fly, *Tipula paludosa* [31,33]	49.8	18.1	1.8	3.9	60	58	17.4		legs	1.4 × 10*^−^*^9^
mosquito, *Aedes aegypti*	1.2	4.4	0.6	0.3	25	470	3.2		legs, body	2.0 × 10*^−^*^12^
woolly aphid, *Eriosomatina*	1.2	3.2	1.2	0.4			2.8		coat, body	1.1 × 10*^−^*^13^
blue-throated hummingbird, *Lampornis clemenciae* [49]	8400	120	20	14	76	23	85			1.0 × 10*^−^*^5^
magnificent hummingbird, *Eugenes fulgens* [49]	7400	130	30	11	75	24	79			1.1 × 10*^−^*^5^
black-chinned hummingbird, *Archilochus alexandri* [49]	3000	83	20	11	63	51	47			1.8 × 10*^−^*^6^
Rufous hummingbird, *Selasphorus rufus* [50,51]	3300	80	15	7	82	52	42	1.2/0.2 [52]		1.8 × 10*^−^*^6^
Mentor SF-2.5 [53]	580 × 10^3^	344	86	83	90	30	180			6.0 × 10*^−^*^3^
DelFly II [54,55]	16 × 10^3^	210	10	20	48	13	140		tail	5.9 × 10*^−^*^5^
Cornell micro-air vehicle I [56,57]	24 × 10^3^	150	15	8	90	24	223		sails	2.0 × 10*^−^*^4^
Cornell micro-air vehicle II [58]	3.9 × 10^3^	30	30	20	90	30	72		sails	4.0 × 10*^−^*^7^
Harvard micro-robotic insect [59]	60	15	5	5	50	110	15			1.2 × 10*^−^*^9^

**Table 2.** Time scales relevant to pitch stability and control for hovering insects, hummingbirds and flapping-wing robots.

	*T*_I_ (ms)	*T*_F_/*T*_I_	*T*_P_/*T*_I_	*T*_INST_/*T*_I_	*T*_RXN_/*T*_I_
honeybee, *Apis mellifera*	26	8.8	20	5.3	0.96
bumblebee, *Bombus terrestris*	27	11	23	5.5	0.92
bumblebee, *Bombus hortorum*	31	7.7	17	5.2	0.87
bumblebee, *Bombus lucorum*	31	7.7	15	5.3	0.88
hover fly, *Episyrphus balteatus*	29	3.8	14	4.7	0.78
drone fly, *Eristaltis tenax*	40	6.3	14	5.1	0.85
hawkmoth, *Manduca sexta* male	45	7.3	7.8	5.8	0.85
hawkmoth, *Manduca sexta* female	44	8.4	6.6	6.3	0.86
fruit fly, *Drosophila melanogaster*	14	5.1	6.1	5.8	0.93
fruit fly with fibres	14	4.0	0.63	—	—
parasitic wasp, *Encarsia formosa*	6	3.7	3.3	7.2	1.2
orchid bee, *Euglossa dissimula*	25	7.6	14	5.3	0.89
orchid bee, *Euglossa imperialis*	25	8.4	16	5.4	0.89
orchid bee, *Eulaema meriana*	37	5.9	11	5.2	0.87
march fly, *Bibio marci* female	34	4.1	7.4	5.3	0.88
march fly, *Bibio marci* male	34	4.4	8.5	5.2	0.86
conopid fly, *Conops strigatus*	31	4.8	9.7	5.1	0.85
bluebottle fly, *Calliphora vicina*	20	8.0	10	5.7	0.94
black fly, *Simulium* Latreille	14	5.2	5.4	6.0	1.0
crane fly, *Tipula paludosa*	27	5.2	4.1	6.8	1.1
mosquito, *Aedes aegypti*	24	3.1	1.8	14.6	2.4
woolly aphid, *Eriosomatina*	15	0.95	0.19	—	—
blue-throated hummingbird, *Lampornis clemenciae*	93	15	19	6.1	1.0
magnificient hummingbird, *Eugenes fulgens*	120	11	23	5.5	0.92
black-chinned hummingbird, *Archilochus alexandri*	75	18	55	6.0	1.0
Rufous hummingbird, *Selasphorus rufus*	89	18	26	6.3	1.0
Mentor SF-2.5	110	11	18	5.7	0.94
DelFly II	140	1.0	0.71	—	—
Cornell micro-air vehicle I	330	0.52	0.20	—	—
Cornell micro-air vehicle II	72	0.93	0.18	—	—
Harvard micro-robotic insect	20	8.0	11	5.6	0.93

As outlined above, the body mass *M*, length *L*, diameter *d* and centre-of-mass to centre-of-drag distance *h* are needed to estimate *T*_I_ · *T*_F_ can be estimated from the body mass *M*, the lift-to-drag ratio *C*_L_/*C*_D_, and rms wing speed at two-thirds span: *w* ≈ *ϕ*_0_(2π*f*)*R*/2, where *ϕ*_0_ is the stroke amplitude, *f* is the wing-beat frequency and *R* is the wing span length. Similarly, *T*_P_ can be calculated from the wing aerodynamic parameters as well as from body morphological parameters. To our knowledge, reliable *C*_L_/*C*_D_ measurements have only been performed recently and are available for the fruit fly, hawkmoth, honeybee and Rufous hummingbird. For other animals and robots, we assume a value that corresponds to one of these animals that is closest in size. Kinematic data are not available for the woolly aphid, so fruit fly parameters have been used.

**Figure 7.**
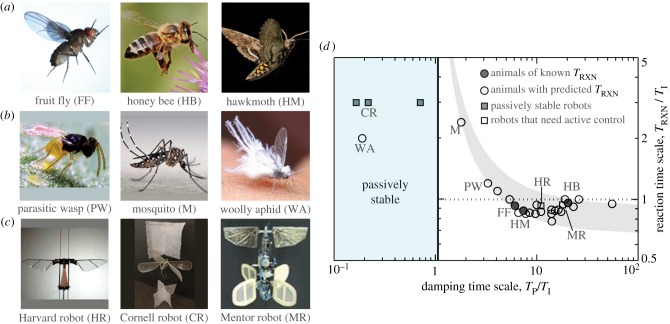
Control requirements for hovering insects, hummingbirds and flapping-wing robots. (*a*) Insects of varying size whose reaction time has been measured or estimated: fruit fly (typical body length 2.7 mm), honeybee (16 mm) and hawkmoth (46 mm). (*b*) Insects with unusual damping characteristics. The viscous drag on the body of the tiny wasp (0.6 mm) is significant, the mosquito (4.4 mm) flies with its long legs extended and the woolly aphid (3.2 mm) has a fibrous coat. (*c*) Robots with different stabilization strategies: Harvard robot (15 mm) is externally stabilized with wire guides; Cornell robot (220 mm) has large sails; Mentor robot (360 mm) uses sensory feedback control. (*d*) Reaction time needed to stabilize flight for hovering animals (circles) and robots (squares). Reaction time is known for the fruit fly, honeybee and hawkmoth (filled circles) and predicted for other flyers (open symbols). Predictions are determined by the rule of thumb that reactions must be six times faster than the instability, with variations within the grey band due to differences in the unplotted parameter *T*_F_/*T*_I_. (Online version in colour.)

In figure 7*a*–*c*, we highlight some animals and machines of varying size and shape, and in figure 2*d* we summarize the stability and control properties of all the flyers tabulated above. In addition to the fruit fly, the honeybee and hawkmoth are important case studies because the flight reaction times of these insects have been measured or estimated. For the honeybee, the reaction time was determined by measuring when compensatory wing motions were induced after a gust perturbation [60]. For the hawkmoth, the allowable delay was estimated in computer simulations of free flight [61]. These insects are represented by filled circles in figure 7*d*, and indeed these data are consistent with the control law that reactions are approximately six times faster than the instability. Thus, these insects have a similar control performance to that of the fruit fly despite their different control systems. In particular, neither the bee nor the moth have halteres, and the hawkmoth probably relies on its antennae for flight stabilization [62]. We are not aware of a study that has identified the relevant sensors for the honeybee.

These findings suggest that our formulation can be generalized to predict the flight control reaction times of other insects. We use the same rule of thumb that reaction time is six times the instability growth time to furnish predictions for a variety of insects, and these data are displayed as open circles in figure 7*d*.

Similarly, there are several prototypes of flapping-wing robots whose stability properties are reported in the literature. In particular, three such robots are designed to be passively stable with the addition of sails or tails that act as rotational dampers. An example is the Cornell robot (CR) shown in figure 7*c*, which relies on fixed sails attached above and below the stroke plane of the wings [58]. This and other damped robots are shown as filled squares in figure 7*d*, and indeed our time scale estimates confirm that the damping is sufficient to passively stabilize flight. Finally, we indicate as open squares the robots that are predicted to be intrinsically unstable and thus require active control. For example, we predict that the Harvard micro-robotic insect (HR) is unstable, which is consistent with the need to restrict its flight along vertical guide wires [59]. Importantly, our formulation furnishes predictions for the controller speed necessary to actively stabilize such designs.

## 5. Discussion

Taken together, these results shed light on the roles played by both physical effects as well as sensory–motor control in the stabilization of insect flight. Our framework unites the elements of stability and control by combining ideas from dynamical systems with control theory. In this respect, our work is part of a growing appreciation of the power of these disciplines in understanding how animals navigate through and manipulate their environment. The spirit of our approach, however, is to apply these techniques while retaining a language that is physically intuitive and biologically meaningful. To this end, our framework is built around the idea that stability and control strategies can be organized by comparing the relevant time scales in the problem.

In the first step of this formulation, we have built upon pioneering simulation work to derive a simplified mathematical model of the body orientation dynamics. Our model shows that the pitch degrees of freedom of an insect body can be described by an equation similar to that of a damped pendulum. Importantly, the pitch dynamics also include a forcing term that is associated with forward motion of the body and that can act to destabilize the pendular motion. Analysis of this model offers a surprisingly simple condition for intrinsic or passive stability: the time scale that characterizes the damping of pitch rotations *T*_P_ must be faster than the time scale that characterizes inertial or pendulum-like oscillations *T*_I_. Thus, intuitively, passive stability requires that damping overcomes body inertia.

The second component of our theory addresses the active control that is needed if this condition is not met and flight is unstable. As our compilation of figure 7*d* shows, most hovering animals rely little on damping and thus are predicted to be intrinsically unstable. In this case, we find a surprisingly simple requirement for achieving well-controlled flight: 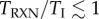, that is, the reaction time must be as fast as the inertial rotation time scale. Intuitively, a flyer with weak damping must apply corrective responses before its body rotates appreciably owing to inertia.

This time-scale formulation offers a natural interpretation of our experiments on the flight performance of fruit flies. By mechanically perturbing the flight of these insects, we find that they indeed react as quickly as their inertial time scale. When we disable the fast sensors, the insects can no longer respond sufficiently quickly and consequently are unable to maintain body orientation during flight. Finally, our formulation shows that, even without fast-acting control, flight could be stabilized if the insect body were modified to increase the degree of pitch damping. We confirm this prediction by attaching high-drag fibres to sensor-disabled fruit flies and observing that they are indeed able to keep upright while aloft.

We envision that this work is but a step in bringing together the elements of stability and control in flapping-wing flight, and each component of our study offers clear avenues for improvement and expansion. First, we have focused on the pitch orientation during normal hovering, a scenario that a variety of models and simulations agree is plagued by a diverging oscillating instability. It may be that other degrees of freedom are also unstable during normal hovering. For example, recent CFD simulations indicate that roll of drone flies exhibits a fast diverging instability [9], though reduced-order aerodynamic models find either slowly growing or decaying oscillatory modes [12,13]. Ultimately, a clear picture of roll stability will probably emerge from the combination of flow simulations, aerodynamic models and experiments, at which point a similar control theory analysis such as the one presented here should be developed. Similarly, future work might examine stability during other flight modes, such as forward motion or ascent, and different wing kinematics, such as the vertical heaving motion of dragonflies. Based upon our study of pitch during normal hovering, we anticipate that the physical stability properties during these other flight conditions will place additional constraints on the sensory–motor systems needed for control.

In spite of these caveats, however, our formulation does allow us to make concrete and testable predictions. For example, our discovery that *T*_RXN_ ≈ *T*_I_ provides a powerful way to estimate sensory–neural control characteristics from morphological factors. Large flyers will generally be able to react more slowly since 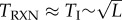, where *L* is the characteristic length scale of the animal or robot. This analysis also shows that small flying insects require fast reaction times. The millimetre-sized parasitic wasp [32], for example, is predicted to need a 7 ms stabilization reflex, which, if validated, would be among the fastest behavioural response times in the animal kingdom [63–65]. Thus, flight control is most challenging at small scales and may be an important factor in determining the lower limit in body size of flying insects.

The strategy of using high-drag surfaces appears to be employed by relatively few insects. For example, the mosquito by extending its long legs in flight provides damping that would enable slower control, as indicated in figure 7*d*. The woolly aphid represents a bizarre extreme in this strategy. Our calculations indicate that its cotton-like fibrous covering provides such strong damping that the aphid may be one of the few passively stable insects.

For biomimetic flapping-wing robots, our time-scale formulation could serve as an important guide in achieving stable flight. Like their biological counterparts, current prototypes can be characterized in terms of passive versus active stabilization. Several implementations that have achieved passive stability rely on the addition of sails or tails which provide damping. For such designs, stability models can be used to determine the size and placement of these surfaces [34,57]. Other robotic designs must rely on active control, and here our finding that the reaction time be at least as fast the inertial rotation time will probably prove to be an important design criterion. In particular, our formulation shows that a key challenge in miniaturizing such designs will be in devising automatic control schemes that can respond quickly enough to keep the device upright.

Finally, we speculate that the strategies used by flying insects may have changed over the course of their 400 million years of evolution. In particular, early flyers are unlikely to have had the fast and sophisticated sensory–neural systems of modern insects and may instead have relied on body plans that confer intrinsic stability [66]. Though the palaeontological record is too incomplete to fully support this conjecture, fossil insects do include damping features such as plate-like lobes and hair-like fibres [67]. The conspicuous absence of damping structures in most modern insects may reflect an adaptation towards manoeuvrability and evasiveness in Nature's increasingly crowded airspace.
